# Permanent Photodynamic Activation of the Cholecystokinin 2 Receptor

**DOI:** 10.3390/biom10020236

**Published:** 2020-02-04

**Authors:** Wen Zhu Tang, Zong Jie Cui

**Affiliations:** Institute of Cell Biology, Beijing Normal University, Beijing 100875, China; twz0810@126.com

**Keywords:** CCK2R, SALPC, miniSOG, calcium oscillations, photodynamic action

## Abstract

The cholecystokinin 2 receptor (CCK2R) is expressed in the central nervous system and peripheral tissues, playing an important role in higher nervous and gastrointestinal functions, pain sensation, and cancer growth. CCK2R is reversibly activated by cholecystokinin or gastrin, but whether it can be activated permanently is not known. In this work, we found that CCK2R expressed ectopically in CHO-K1 cells was permanently activated in the dark by sulfonated aluminum phthalocyanine (SALPC/AlPcS_4_, 10–1000 nM), as monitored by Fura-2 fluorescent calcium imaging. Permanent CCK2R activation was also observed with AlPcS_2_, but not PcS_4_. CCK2R previously exposed to SALPC (3 and 10 nM) was sensitized by subsequent light irradiation (>580 nm, 31.5 mW·cm^−2^). After the genetically encoded protein photosensitizer mini singlet oxygen generator (miniSOG) was fused to the N-terminus of CCK2R and expressed in CHO-K1 cells, light irradiation (450 nm, 85 mW·cm^−2^) activated in-frame CCK2R (miniSOG-CCK2R), permanently triggering persistent calcium oscillations blocked by the CCK2R antagonist YM 022 (30 nM). From these data, it is concluded that SALPC is a long-lasting CCK2R agonist in the dark, and CCK2R is photogenetically activated permanently with miniSOG as photosensitizer. These properties of SALPC and CCK2R could be used to study CCK2R physiology and possibly for pain and cancer therapies.

## 1. Introduction

The G protein-coupled cholecystokinin type 2 receptor (CCK2R) is widely distributed in the central nervous system (CNS) [1,2]. CCK2R in the CNS regulates food intake and satiety [3,4]. CCK2R is involved in anxiety and panic disorders and is correlated with suicidal behavior [2,5,6,7]. In the periphery, CCK2R is expressed in gastrointestinal tissues [8,9,10,11]. Gastrin stimulation of CCK2R promotes gastric acid secretion [12], inhibits stomach emptying [13], inhibits colon motility [14], and in the long term promotes the branching morphogenesis of gastric epithelial cells [15].

Evidence for the involvement of CCK2R in pain sensitization/perception is abundant. For example, CCK2R has been confirmed as being expressed in dorsal root ganglion neurons to mediate the inhibition of a Kv current (I_A_) to increase pain sensation [16]. The CCK2R antagonist is known to reduce the need for opioid administration in relieving pain in a mouse model of burn-induced pain [17], likely by antagonizing CCK2R–μ-opioid receptor (MOR) heterodimerization, which inhibits MOR signaling [18]. CCK infusion into amygdala in rats has been found to activate a descending CCK2R-mediated pathway and to inhibit spinal neuron discharge, leading to hindpaw analgesia [19]. CCK2R knockout is known to relieve pain in mouse models of chronic constrictive injury [20].

Further, CCK2R is overexpressed in cancers of the thyroid, lungs, ovaries, and gastrointestinal tract and in neuroendocrine tumors and gliomas [21,22,23,24,25], where CCK2R is known to promote cancer cell growth and proliferation [24,26].

CCK2R is typically coupled to the G protein type q (Gq) [26,27]. The stimulation of CCK2R expressed in cell lines has been found to be associated with calcium mobilization and the appearance of calcium oscillations; therefore, calcium signaling is a useful indicator of CCK2R activation [28,29].

CCK receptor activation has been found to be associated with changes in receptor oligomerization status, but clear differences have been noted between CCK1R and CCK2R. CCK1R is known to form dimers or oligomers, which dissociate into monomers upon agonist stimulation, but CCK2R dimers or oligomers are not affected by agonist stimulation [30]. On the contrary, agonist stimulation of coexpressed CCK1R and CCK2R has been found to form CCK1R-CCK2R heterodimers [29]. Further, the heterodimerization of CCK2R and the μ-opioid receptor (MOR) has been found to reduce or antagonize opioid agonist-induced MOR activation, downstream signaling, and MOR analgesia [18]. Although agonist activation and associated changes in CCK receptor oligomerization status are interesting, the detailed chemical basis for such activation is lacking. Interestingly, it has recently been found that the CCK1 receptor (CCK1R) could be readily activated by the lowest-lying excited state of molecular oxygen (i.e., the delta singlet oxygen, ^1^O_2_) in the complete absence of any agonist, as described below.

^1^O_2_ is typically generated in a type II photodynamic action after photosensitizer absorption of a photon at an appropriate wavelength [31,32]. One widely used class of chemical photosensitizers is phthalocyanines (Pc) [33]. Macrocyclic phthalocyanines with centrally conjugated metal ions (such as Zn, Al, or Si) are highly efficient generators of ^1^O_2_ [34]. The peripheral functionalization of phthalocyanines is normally needed for aqueous solubility and reduced aggregation [35,36]. Four peripherally functionalized phthalocyanine photosensitizers are in common use in clinical medicine: Photoens, Pc 4, CGP55847 (ZnPc), and Photocyanine [35]. Photoens^®^ is an aqueous solution of sulfonated aluminum phthalocyanine (SALPC or AlPcS_4_) [35]. SALPC or AlPcS_4_ with a central conjugated Al^3+^ and four peripheral sulfonate groups is an efficient photosensitizer with good aqueous solubility and a high ^1^O_2_ quantum yield [36,37].

SALPC becomes bound to the plasma membrane of freshly isolated rat pancreatic acini after a brief (10 min) incubation: the subsequent light irradiation of bound SALPC (i.e., SALPC photodynamic action) has been found to permanently activate CCK1R, triggering persistent calcium oscillations blocked by the CCK1R antagonist FK-480 [38,39]. Intrinsically expressed CCK1R in the rat pancreatic acinar tumor cell AR4-2J and heterologously expressed CCK1R in HEK293 cells are similarly activated permanently through SALPC photodynamic action [40]. Other than the phthalocyanine photosensitizer SALPC, the genetically encoded protein photosensitizers KillerRed or mini singlet oxygen generator (miniSOG) have also been found to permanently activate CCK1R photodynamically after light irradiation [41]. Our most recent work identified the third transmembrane (TM3) domain of CCK1R, especially the YxM oxidative motif, as the most likely pharmacophore for photodynamic or photogenetic activation of CCK1R with fused KillerRed or miniSOG [42]. An examination of the TM3 domains of CCK1R and CCK2R could identify a high homology sequence of KxxxYxMGxSVSVSTxxLVAIxLER (where x represents any amino acid residue), note especially the presence of the YxM oxidative motif in CCK2R [42].

On the basis of the above reports, in the present work we investigated the photodynamic modulation of CCK2R, with both SALPC and miniSOG as photosensitizers. Remarkably, it was found that SALPC in the dark could permanently activate CCK2R ectopically expressed in CHO-K1 cells; light irradiation only further enhanced this activation. In addition, it was found that miniSOG fused to the N-terminus of CCK2R (as a construct of miniSOG–CCK2R) photodynamically activated in-frame CCK2R after light irradiation. These novel CCK2R-activating properties of SALPC could be explored further in the future both in the study of CCK2R physiology and in the study of potential pain or cancer therapies. The photodynamic or photogenetic activation of CCK2R with miniSOG or other genetically encoded protein photosensitizers would have immediate implications for potential in vivo studies of CCK2 receptor physiology both in the CNS and in peripheral tissues.

## 2. Materials and Methods

### 2.1. Materials

The sulfated cholecystokinin octapeptide (CCK) and the CCK2R antagonist YM 022 (C_32_H_28_N_4_O_3_) were from Tocris Cookson (Bristol, UK). Dulbecco’s Modified Eagle’s Medium (DMEM)/F12 (1:1), trypsin 0.25% were from Invitrogen (Shanghai, China). Goat anti-CCK2R antibody and tetramethylrhodamine isothiocyanate (TRITC)-conjugated rabbit antigoat secondary antibody were from Abcam (Cambridge, UK). Hoechst 33342 was from DojinDo (Beijing, China). Top10 competent cells, ampicillin, horseradish peroxidase (HRP)-conjugated goat antimouse IgG, and HRP-conjugated rabbit antigoat IgG were from Beijing Kangweishiji (Beijing, China). Plasmid *pcDNA3.1+/CCK2R* (CCK2R GenBank accession number AY322551) was from the cDNA Resource Center (cDNA.org, originally in Rolla, MO, but now in Bloomsburg, PA, USA). Plasmid with a plasma membrane (PM)-localizing sequence, *pKillerRed_PM_*, was bought from Evrogen (cat. no. FP966, Moscow, Russia). Sulfonated aluminum phthalocyanine (SALPC or AlPcS_4_, C_32_H_16_AlClN_8_O_12_S_4_), PcS_4_ (phthalocyanine tetrasulfonic acid, C_32_H_18_N_8_O_12_S_4_), and AlPcS_2_ [Al(III) phthalocyanine chloride disulfonic acid, C_32_H_16_AlClN_8_O_6_S_2_] were from Frontier Scientific (West Logan, UT, USA). In addition, 4-(2-hydroxyethyl)-1-piperazineethanesulfonic acid (HEPES) was from Calbiochem (Darmstadt, Germany). Fura-2 AM (ultrapure grade) was from AAT Bioquest (Sunnyvale, CA, USA). Cell-Tak was from BD Bioscience (Bedford, MA, USA). Endotoxin-free plasmid extraction kit was from TianGen Biochemicals (Beijing, China). The JetPRIME^®^ transfection reagent was from Polyplus-Transfection (Illkirch, France).

### 2.2. Vector Constructs and CHO-K1 Cell Transfection

A mammalian codon-optimized *miniSOG* gene (GenBank accession number JX999997) was synthesized from nucleotides by Genscript (Nanjing, China). The *miniSOG* sequence used was ATGGAAAAGAGCTTTGTGATTACCGATCCGCGCCTGCCAGACAACCCGATCATTTTCGCGAGCGATGGCTTTCTGGAGTTAACCGAATATTCTCGTGAGGAAATTCTGGGTCGCAATGGCCGTTTCTTGCAGGGTCCGGAAACGGATCAAGCCACCGTGCAGAAAATCCGCGATGCGATTCGTGACCAACGCGAAATCACCGTTCAGCTGATTAACTATACGAAAAGCGGCAAGAAATTTTGGAACTTACTGCATCTGCAACCGATGCGCGATCAGAAAGGCGAATTGCAATATTTCATTGGTGTGCAGCTGGATGGCTAG. This *miniSOG* sequence was inserted into the plasmid *pKillerRed_PM_* (Evrogen, Moscow, Russia) to replace the *KillerRed* sequence and to obtain *pminiSOG_PM_*. For the construction of recombinant plasmid *pminiSOG-CCK2R*, the *miniSOG* sequence was amplified from plasmid *pminiSOG_PM_*, and the amplified fragment was cloned and ligated to the N-terminal of CCK2R in vector *pcDNA3.1+/CCK2R* (by Genscript (Nanjing, China)).

The plasmids, thus obtained, were used to transfect the CHO-K1 cells. A predetermined amount of each plasmid (2 μg DNA) was mixed in transfection buffer (200 μL) before addition of transfection reagent (jetPRIME^®^, 4 μL of stock). After thorough mixing, the mixture was allowed to sit for 10 min at room temperature before use. CHO-K1 cells were planted in each culture plate (35 mm), and 24 h later, 200 μL of the above transfection mixture (containing both the plasmids and the transfection reagent jetPRIME^®^) was added. Transfected CHO-K1 cells were used for the measurement of cytosolic calcium concentration and immunocytochemistry 24 h after transfection.

### 2.3. Cell Culture (CHO-K1, Escherichia coli)

Chinese hamster ovary K1 (CHO-K1) cells were purchased from the Shanghai Institute of Life Sciences Chinese Academy of Sciences and cultured in DMEM/F12 (1:1) supplemented with 10% fetal bovine serum (FBS, Gibco, Shanghai, China) in a CO_2_ incubator under 5% CO_2_ at 37 °C.

A solid *E. coli* medium LB/Amp was sterilized and made into culture plates. The liquid *E. coli* medium LB/Amp had the same composition but without agar.

### 2.4. Immunocytochemistry

Dispersed cells were attached to Cell-Tak-coated coverslips before fixation in paraformaldehyde 4% (10 min). Cells were permeabilized in 0.2% Triton X-100 in phosphate-buffered saline (PBS) for 15 min and then washed. Nonspecific binding was blocked in 3% bovine serum albumin (BSA) in PBS for 1 h before incubation with a primary antibody (1:100) in a humid chamber at 4 °C overnight, followed by incubation with secondary antibody (30 min). The cells were then counterstained with Hoechst 33342 for 15 min and washed. The washes (after incubation with primary and secondary antibodies and with Hoechst) were all done in PBS containing 0.2% Triton X-100 and 2% Tween-20. The cell-attached coverslips were placed on glass slides, sealed, and stored at 4 °C in the dark before being imaged in a confocal microscope (Zeiss LSM 510 META, objective 63×/1.40 oil) (Oberkochen, Germany).

### 2.5. Measurement of Cytosolic Calcium Concentration

Cells were loaded with Fura-2 AM (final concentration 10 μM) at 37 °C in a shaking water bath at 50 cycles/min for 30 min. Fura-2-loaded cells were allowed to attach to coverslips previously coated with Cell-Tak (0.6 g·L^−1^, 1 μL on each coverslip) that formed the bottom part of Sykes–Moore perfusion chambers. Cell attachment was allowed for at least 30 min before perifusion and experimentation.

A cell-attached perfusion chamber was placed on the platform of an inverted fluorescence microscope (Nikon NE 3000) connected to a calcium detection system (Photon Technology International, PTI, Edison, NJ, USA). Fura-2 was excited alternately at 340 nm and 380 nm (monochromator DeltaRam X), and the emitted fluorescence was imaged at 510 nm (510 ± 40 nm) with CCD (NEO-5.5-CL-3, Andor/Oxford Instruments, Belfast, UK). The fluorescence ratios F_340_/F_380_ indicative of cytosolic calcium concentrations were plotted against time with SigmaPlot, as reported previously [40,41].

### 2.6. Statistical Analyses

All data are presented as mean ± SEM (standard error of means). A Student’s *T*-test was done, and statistically significant differences are indicated by an asterisk (*) at *p* < 0.05.

## 3. Results

### 3.1. CCK2R Expression in CHO-K1 Cells

**Figure 1:**
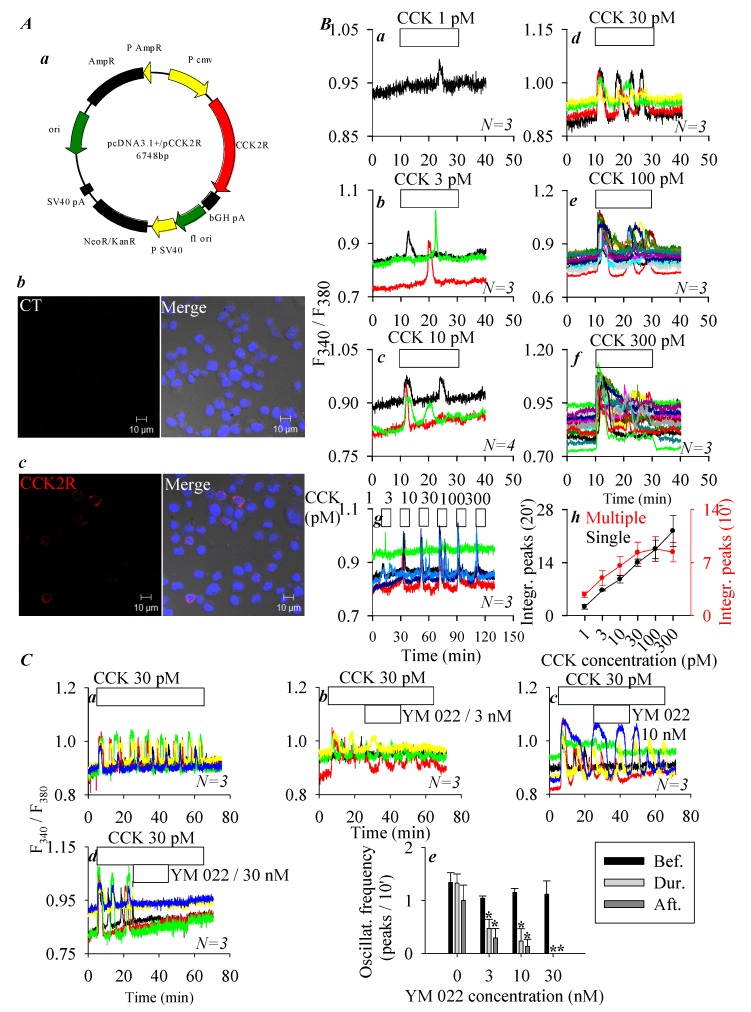
Ectopic expression of cholecystokinin 2 receptors (CCK2R) in CHO-K1 cells. (**A**) CHO-K1 cells were transfected with plasmid *pCCK2R* (**Aa**), and 48 h after transfection, CCK2R-CHO-K1 cells were fixed and attached to coverslips, incubated sequentially with primary goat anti-CCK2R antibody and TRITC-conjugated donkey antigoat secondary antibody (red), and counterstained with nuclear dye (Hoechst 33342, blue) before confocal imaging (**Ac**). Shown here are fluorescent and merged brightfield images. The controls (with only secondary antibody) done in CCK2R-CHO-K1 cells did not show any fluorescence (**Ab**). Confocal images were taken in a Zeiss LSM 510 META (objective 63×/1.40 oil) with λ_ex_: TRITC/543 nm and Hoechst 33342/405 nm. Scale bars 10 μm. (**B**) CCK2R-CHO-K1 cells loaded with Fura-2 AM were attached to coverslips forming the bottom part of a Sykes–Moore perfusion chamber, perifused, and exposed to CCK at 1 (**Ba**), 3 (**Bb**), 10 (**Bc**), 30 (**Bd**), 100 (**Be**), and 300 pM (**Bf**) as indicated by the horizontal bars. (**Bg**) CCK2R-CHO-K1 cells were stimulated sequentially with CCK at 1, 3, 10, 30, 100, and 300 pM. (**Bh**) A quantitative analysis was performed, and the calcium peak areas above the baseline in (**Ba–Bf**) were calculated (from 10–30 min, black curve). The calcium peak areas above the baseline in (**Bg**) were calculated for 10 min periods (10–20, 30–40, 50–60, 70–80, 90–100, and 110–120 min, red curve). (**C**) CCK2R-CHO-K1 cells loaded with Fura-2 AM were attached to the coverslip bottoms of Sykes–Moore perfusion chambers, perifused, and exposed to CCK 30 pM and YM 022 at 0 (**Ca**), 3 (**Cb**), 10 (**Cc**), and 30 nM (**Cd**), as indicated. (**Ce**) A quantitative analysis was done for the original tracings shown in (**Ca–Cd**); the number of calcium peaks per 10 min before, during, and after perfusion of YM 022 is presented. Student’s *T*-test was performed, statistically significant difference is indicated by an asterisk (*) at *p* < 0.05. Representative calcium tracings from *N* identical experiments (*N* = 3–4) are shown in *(***Ba**–**Bg**, **Ca**–**Cd**).

Heterologous CCK2R expression in CHO-K1 cells (which express no endogenous CCK2R) has previously been reported to result in a fully functional receptor that upon activation by CCK triggers calcium increases via the Gq signaling pathway [28,29]. In the present work, CCK2R was transiently expressed in CHO-K1 cells, immunocytochemistry 48 h after transfection revealed plasma membrane expression of CCK2R (Figure 1A). No fluorescence was detected in control cells without previous incubation with primary antibody but with secondary antibody (Figure 1A). CCK (1, 3, 10, 30, 100, and 300 pM) concentration-dependently stimulated calcium increases in CCK2R-CHO-K1 cells (Figure 1(Ba)–(Bf)): this concentration dependence in different cells (Figure 1(Ba)–(Bf)) was also found in the same CCK2R-CHO-K1 cells (Figure 1(Bg)). The quantification of calcium peaks revealed a monophasic dose–response curve for CCK stimulation either in separate cells or in the same cells: in the same cells a tendency to desensitize was apparent (Figure 1(Bh)).

CCK 30 pM-induced calcium oscillations in CCK2R-CHO-K1 cells were dose-dependently inhibited by the CCK2R antagonist YM 022 (3, 10, 30 nM, Figure 1C). YM 022 at 30 nM was able to completely inhibit calcium oscillations induced by CCK 30 pM in CCK2R-CHO-K1 cells (Figure 1(Cd)). The frequency of calcium oscillations was calculated for before, during, and after the addition of different YM 022 concentrations. Very clear antagonist-concentration-dependent inhibition of CCK-induced calcium oscillations was observed (Figure 1(Ce)). These data clearly indicate that after expression in CHO-K1 cells CCK2R was coupled to the calcium signaling pathway, consistent with previous reports in the literature [28,29].

### 3.2. Permanent Activation of CCK2R by SALPC and an Analogue in the Dark

**Figure 2:**
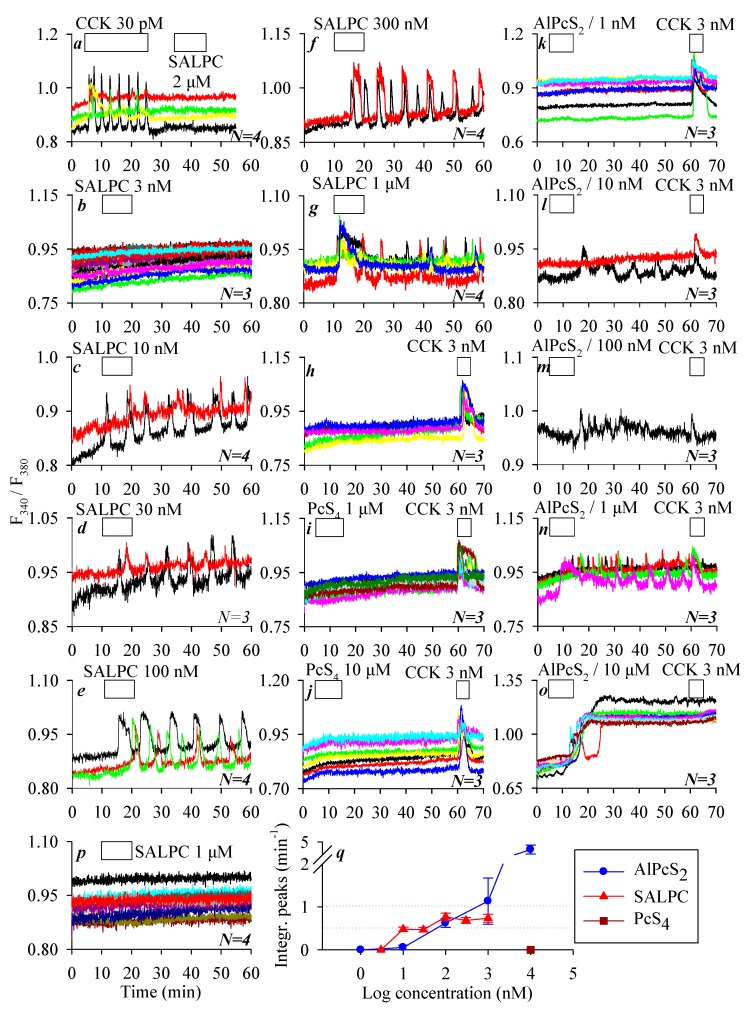
Phthalocyanine molecules SALPC (AlPcS_4_) and AlPcS_2_, but not PcS_4_ activated CCK2R in the dark to trigger calcium oscillations in CCK2R-CHO-K1 cells. CCK1R-CHO-K1 (**a**), CCK2R-CHO-K1 (**b**–**o**), and CHO-K1 (**p**) cells loaded with Fura-2 AM were attached to coverslips forming the bottom part of Sykes–Moore chambers and perifused. CCK 30 pM and SALPC 2 μM (**a**), SALPC 3 nM (**b**), 10 nM (**c**), 30 nM (**d**), 100 nM (**e**), 300 nM (**f**), or 1000 nM (**g**); CCK 3 nM (**h**); PcS_4_ 1 μM (**i**); PcS_4_ 10 μM (**j**); AlPcS_2_ 1 nM (**k**); AlPcS_2_ 10 nM (**l**); AlPcS_2_ 100 nM (**m**); AlPcS_2_ 1000 nM (**n**); or AlPcS_2_ 10,000 nM (**o**) plus CCK 3 nM (**i**–**o**), or SALPC 1 µM (**p**) were added as indicated by the horizontal bars. Note the lack of any effect of SALPC 1 µM on parental CHO-K1 cells not transfected with CCK2R (**p**). Quantitative analysis was done for the experiments shown in (**b**–**o**), and the calculated peak area above baseline is presented (**q**). Representative calcium tracings from one of *N* identical experiments (*N* = 3–4) are shown in (**a**–**p**).

We have previously found that the chemical photosensitizer SALPC (i.e., AlPcS_4_) had no effect on CCK1 receptors in the dark at micromolar concentrations. Indeed, in the present work, we could confirm that SALPC at 2 μM in the dark had no effect on the baseline calcium concentration in CCK1R-CHO-K1 cells, whereas CCK at 30 pM induced robust calcium oscillations (Figure 2a). In contrast, SALPC induced persistent calcium oscillations in CCK2R-CHO-K1 cells, with very clear concentration dependence: SALPC 3 had no effect, but 10–1000 nM all induced persistent calcium oscillations lasting until the end of the experiments after SALPC was washed out (Figure 2b–g). The minimum effective SALPC concentration was 10 nM, SALPC of 30, 100, and 300 nM all induced very regular calcium oscillations, whereas SALPC 1000 nM induced an initial phasic increase that decayed into oscillatory increases (Figure 2c–g). The integration of the calcium peak areas above baseline after SALPC addition showed a very clear SALPC dose–response effect (Figure 2q).

To shed light on the structure–function relationship, the effects of two structural analogues of SALPC, PcS_4_ and AlPcS_2_, were examined in CCK2R-CHO-K1 cells. In parallel experiments, CCK2R-CHO-K1 cells showed a stable baseline calcium level in resting conditions, but CCK 3 nM induced ready calcium response at the end of the experiment (Figure 2h). The SALPC analogue PcS_4_ elicited no calcium response at rather high concentrations of 1 μM and 10 μM in CCK2R-CHO-K1 cells, which showed a ready response to stimulation with CCK 3 nM (Figure 2i,j). A second SALPC analogue, AlPcS_2_, in contrast, induced persistent calcium oscillations in a concentration-dependent manner (1, 10, 100, 1000, and 10,000 nM) in CCK2R-CHO-K1 cells. AlPcS_2_ at 1 nM had no effect on the baseline calcium concentration; the fact that CCK 3 nM induced ready calcium response at the end of the experiment confirmed the presence of functional CCK2R in the CCK2R-CHO-K1 cells (Figure 2k). The minimum effective AlPcS_2_ concentration was found to be 10 nM, which induced sparse calcium spikes in some cells; CCK 3 nM at the end of the experiment induced additive calcium increases in all of the CCK2R-CHO-K1 cells examined (Figure 2l). AlPcS_2_ (100 nM and 1 µM) also induced persistent calcium oscillations in CCK2R-CHO-K1 cells, with the end-of-experiment additive response with CCK 3 nM still present (Figure 2m,n). AlPcS_2_ at 10 µM induced an initial oscillatory response followed by an elevated calcium plateau, upon which CCK 3 nM induced no additional calcium increase (Figure 2o). In parallel experiments, it was found that SALPC 1 µM had no effect on the baseline calcium concentration in parental CHO-K1 cells not transfected with CCK2R (Figure 2p), in sharp contrast to the strong calcium oscillations induced by SALPC 1 µM in CCK2R-CHO-K1 cells (Figure 2g). The integrated calcium responses (peak areas above baseline) induced by different concentrations of AlPcS_2_ are also shown (Figure 2q). Note that of the three phthalocyanines (SALPC, PcS_4_, and AlPcS_2_), PcS_4_ was completely ineffective. The order of potency of the other two phthalocyanines was SALPC > AlPcS_2_ at concentrations ≤100 nM, but AlPcS_2_ > SALPC at concentrations ≥300 nM (Figure 2q).

### 3.3. SALPC Photodynamic Sensitization of CCK2R in CCK2R-CHO-K1 Cells

**Figure 3:**
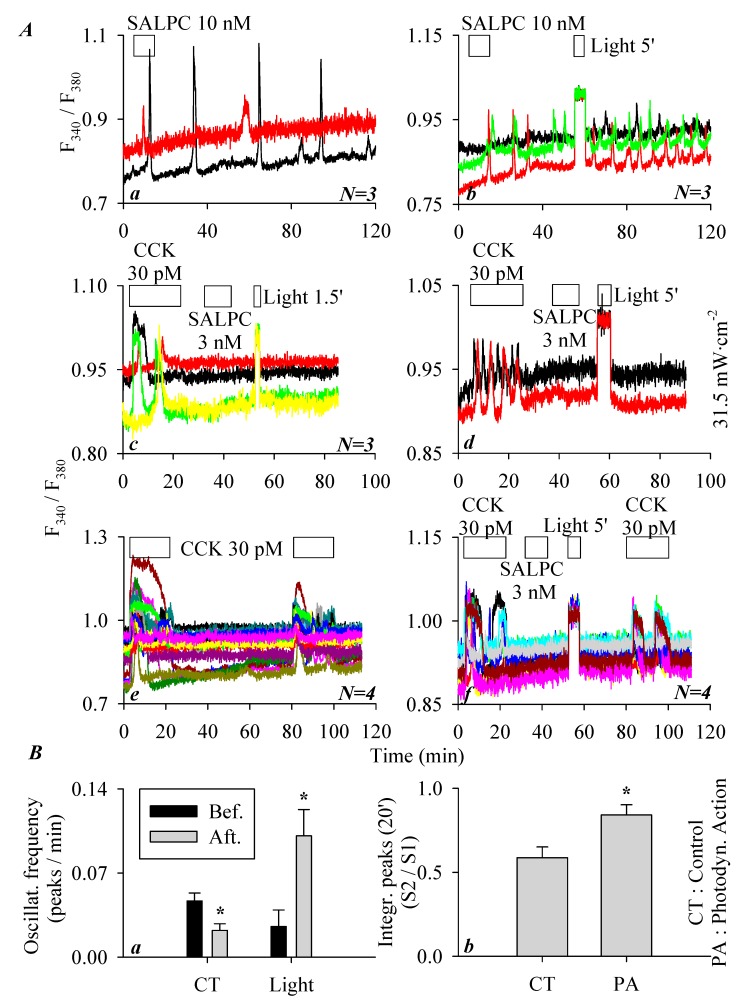
SALPC photodynamic sensitization of CCK2R in CCK2R-CHO-K1 cells. CCK2R-CHO-K1 cells loaded with Fura-2 AM were attached and perifused. CCK 30 pM and SALPC 3 or 10 nM and red light irradiation (λ > 580 nm, 31.5 mW·cm^−2^) were applied as indicated by the horizontal bars. (**Aa**) SALPC 10 nM, no light. (**Ab**) SALPC 10 nM, red light (>580 nm, 31.5 mW·cm^−2^, 5 min). (**Ac**) CCK 30 pM, SALPC 3 nM, red light (λ > 580 nm, 31.5 mW·cm^−2^, 90 s). (**Ad**) CCK 30 pM, SALPC 3 nM, red-light irradiation (λ > 580 nm, 31.5 mW·cm^−2^, 5 min). (**Ae**) CCK 30 pM. (**Af**) CCK 30 pM, SALPC 3 nM, red-light irradiation (λ > 580 nm, 31.5 mW·cm^−2^, 5 min), CCK 30 pM. (**Ba**) The number of calcium peaks per unit time (calcium oscillation frequency) before illumination (5–55 min, Bef.), after illumination (60–110 min, Aft.), or during corresponding time periods without illumination was quantified. (**Bb**) The ratio of the calculated peak area above baseline (S2/S1 of CCK stimulation) in (**Ae**,**Af**) is presented. Student’s *t*-test was done, statistically significant difference is indicated by an asterisk (*) at *p* < 0.05. Representative calcium tracings from one of *N* identical experiments (*N* = 3–4) are shown in (**Aa**–**Af**).

As described above, the minimum effective SALPC concentration to activate CCK2R and therefore induce persistent calcium oscillations in CCK2R-CHO-K1 cells was 10 nM (Figure 2c). To examine any possible photodynamic effect of SALPC, the following series of experiments was performed. In parallel experiments, it was found that SALPC 10 nM induced persistent calcium oscillations in CCK2R-CHO-K1 cells (Figure 3(Aa)). Although the calcium oscillations induced by SALPC 10 nM were long-lasting, red-light illumination (>580 nm, 31.5 mW·cm^−2^, 5 min) markedly increased the oscillatory frequency (Figure 3(Ab)). A quantification of calcium responses, as shown in the representative calcium tracings in Figure 3(Aa,Ab) (calcium oscillation frequency for 50 min before and after light illumination, in peaks/min), revealed that with time, SALPC 10 nM-induced calcium oscillations tended to desensitize, but after light irradiation (>580 nm, 31.5 mW·cm^−2^, 5 min), this desensitization was reversed, and instead sensitization was observed (Figure 3(Ba)). Therefore, SALPC photodynamic action tended to sensitize the activated CCK2R to elicit more frequent calcium oscillations.

As described above, SALPC at 3 nM (in the dark) had no effect on baseline calcium in CCK2R-CHO-K1 cells, and the possible photodynamic modulation was also examined with red-light illumination (>580 nm, 31.5 mW·cm^−2^, 90 s). In this series of experiments, it was found that although CCK 30 pM induced a ready calcium response in CCK2R-CHO-K1 cells, the baseline calcium was not altered by the addition of SALPC 3 nM (Figure 3(Ac)). Further, red-light irradiation (1.5 min) induced no changes in calcium concentration (Figure 3(Ac)). Extended red-light irradiation (5 min) also induced no changes in baseline calcium concentration (Figure 3(Ad)).

In an additional series of experiments, CCK2R-CHO-K1 cells were exposed to tandem doses of CCK 30 pM. Both doses of CCK 30 pM induced regular calcium oscillations, but the second response seemed smaller (desensitization) (Figure 3(Ae)). If in between the two doses of CCK 30 pM, photodynamic action was introduced (with SALPC 3 nM and subsequent light irradiation at >580 nm, 31.5 mW·cm^−2^, 5 min), the second dose of CCK 30 pM also triggered calcium oscillations (Figure 3(Af)). A comparison of the ratio of the two CCK stimulations (S2/S1, in Figure 3(Ae,Af)) revealed that SALPC photodynamic action (SALPC 3 nM, λ > 580 nm, 31.5 mW·cm^−2^, 5 min) significantly sensitized the second dose of CCK stimulation (Figure 3(Bb)). SALPC photodynamic action at SALPC 3 nM therefore did not activate CCK2R, but SALPC photodynamic action did sensitize CCK2R either in a state activated by SALPC 10 nM or in a resting state (SALPC 3 nM) between two doses of CCK 30 pM stimulation.

Due to the inherent bioactivity of SALPC toward CCK2R in the dark even at a relatively low concentration of 10 nM, it was advisable to use alternative photosensitizers to further examine the photodynamic modulation of CCK2R. In the following section, we describe our investigation of the photodynamic action of the genetically encoded protein photosensitizer miniSOG.

### 3.4. miniSOG_PM_ Photogenetic/Photodynamic Activation of CCK2R in miniSOG-CCK2R-CHO-K1 Cells

**Figure 4:**
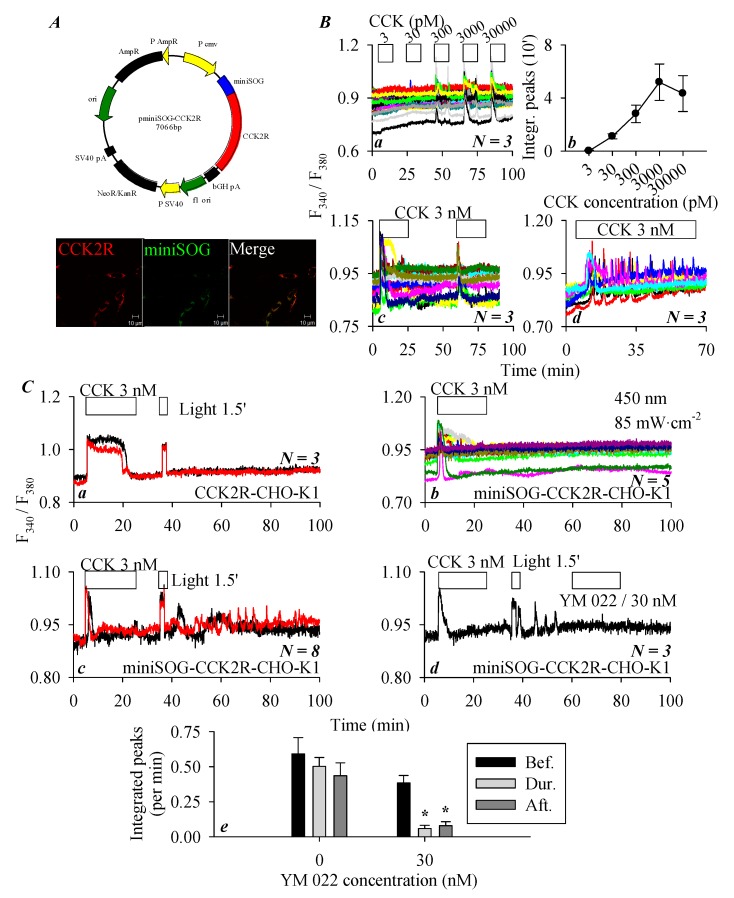
The miniSOG photodynamic activation of CCK2R in CCK2R-CHO-K1 cells. (**A**) CHO-K1 cells were transfected with plasmid *pminiSOG-CCK2R* (**Aa**). Twenty four (24) h after transfection, miniSOG-CCK2R-CHO-K1 cells were fixed and incubated sequentially with primary goat anti-CCK2R antibody and TRITC-conjugated donkey antigoat secondary antibody (red), before confocal imaging done (**Ab**). Fluorescent and merged brightfield images are shown. Confocal images were taken on a Zeiss LSM 510 META (objective 63×/1.40 oil) with λ_ex_ TRITC/543 nm and λ_ex_ 488 nm. Scale bars are 10 μm. (**B**) Here, miniSOG-CCK2R-CHO-K1 or CCK2R-CHO-K1 cells loaded with Fura-2 AM were attached to coverslips of Sykes–Moore perfusion chambers and perifused. CCK at 3, 30, 300, 3000, or 30,000 pM, blue-light irradiation (450 nm, 85 mW·cm^−2^), and YM 022/30 nM were applied as indicated by the horizontal bars. (**Ba**) CCK at 3, 30, 300, 3000, or 30,000 pM. (**Bb**) A quantitative analysis was done for the experiments shown in (**Ba**): the calcium peak areas above baseline were calculated and plotted in terms of CCK stimulation per 10 min (5–15, 25–35, 45–55, 65–75, and 85–95 min). (**Bc**) Tandem doses of CCK 3 nM. (**Bd**) Continuous CCK at 3 nM (1 h); (**C**) miniSOG photodynamic activation of CCK2R. (**Ca**) CCK 3 nM and blue-light irradiation (450 nm, 85 mW·cm^−2^, 90 s) in CCK2R-CHO-K1 cells. (**Cb**–**Cd**) CCK 3 nM (**Cb**), CCK 3 nM, and blue-light irradiation (450 nm, 85 mW·cm^−2^, 90 s) in miniSOG-CCK2R-CHO-K1 cells (**Cc**). CCK 3 nM, blue-light irradiation (450 nm, 85 mW·cm^−2^, 90 s), and YM 022/30 nM in miniSOG-CCK2R-CHO-K1 cells (**Cd**). (**Ce**) Quantitative analysis was performed for the calcium tracings shown in (**Cc**,**Cd**), calcium peak area above the baseline per unit time before, during, and after perfusion with YM 022 was calculated. Student’s *t*-test was done, and statistically significant difference is indicated by an asterisk (*) at *p* < 0.05. Representative calcium tracings from one of *N* identical experiments (*N* = 3–8) are shown in (**Ba**,**Bc**,**Bd**,**Ca**–**Cd**).

To examine the miniSOG photodynamic modulation of CCK2R, a miniSOG-CCK2R fusion protein was designed. The plasmid construct *pminiSOG-CCK2R* (Figure 4(Aa)) or a separate vector, *pCCK2R*, was transduced into CHO-K1 cells that expressed no intrinsic CCK2R. Immunocytochemistry revealed the clear plasma membrane colocalization of CCK2R and miniSOG in miniSOG-CCK2R-CHO-K1 cells (Figure 4(Ab)). In those miniSOG-CCK2R-CHO-K1 cells, sequential doses of CCK (3, 30, and 300 pM and 3 and 30 nM) induced concentration-dependent increases in cytosolic calcium concentration (Figure 4(Ba)). CCK 3 pM was found to have no apparent effect on basal calcium, CCK 30 pM induced a calcium increase but sparingly. The minimum effective CCK concentration seemed to be 300 pM in the majority of the miniSOG-CCK2R-CHO-K1 cells, and CCK 3 and 30 nM produced stronger calcium increases (Figure 4(Ba)). Quantified calcium responses (integrated peak areas) revealed a clear concentration–response curve, which seemed to be bell-shaped, with a maximum concentration of 3 nM (Figure 4(Bb)). In addition, tandem doses of CCK 3 nM resulted in reproducible calcium responses in miniSOG-CCK2R-CHO-K1 cells (Figure 4(Bc)), continuous stimulation by CCK 3 nM produced regular calcium oscillations (with some desensitization at prolonged time points) (Figure 4(Bc,Bd)). These data, altogether, indicate that CCK2R function was retained in the fusion construct of miniSOG-CCK2R, although CCK efficacy was much reduced.

In separate experiments, it was found that CCK 3 nM elicited a robust calcium response (a calcium plateau) in CCK2R-CHO-K1 cells expressing the stand-alone CCK2R, but blue-light irradiation (450 nm, 85 mW·cm^−2^, 90 s) had no effect, indicating that light irradiation had no effect in the absence of miniSOG (Figure 4(Ca)). On the other hand, CCK 3 nM triggered calcium oscillations in miniSOG-CCK2R-CHO-K1 cells, as expected; the wash-out of CCK led to the immediate cessation of CCK-induced calcium oscillations, followed by a stable baseline calcium concentration over an extended time period (Figure 4(Cb)). However, if after CCK stimulation and wash-out of CCK-induced calcium oscillations blue LED-light irradiation (450 nm, 85 mW·cm^−2^, 90 s) was applied, persistent calcium oscillations emerged in those miniSOG-CCK2R-CHO-K1 cells expressing the in-frame miniSOG (Figure 4(Cc)). These LED-light-irradiation-induced persistent calcium oscillations in miniSOG-CCK2R-CHO-K1 cells were blocked completely by the CCK2R antagonist YM 022 (30 nM) (Figure 4(Cd)). Calculation of the integrated calcium peaks revealed that the LED-light-induced calcium oscillations remained stable over a relatively long period of time (left part of the panel, without YM 022), but after the addition of the CCK2R antagonist YM 022 (30 nM), LED-light-induced calcium oscillations were completely inhibited (right part of the panel with YM 022 at 30 nM) (Figure 4(Ce)). From the above data, it is clear that miniSOG in construct miniSOG-CCK2R could photodynamically activate rather permanently the in-frame CCK2R in miniSOG-CCK2R-CHO-K1 cells.

As shown above, CCK2R affinity toward CCK seemed to be much reduced in miniSOG-CCK2R-CHO-K1 cells in comparison to CCK2R-CHO-K1 cells. A comparison of CCK dose–response curves of CCK2R-CHO-K1 and miniSOG-CCK2R-CHO-K1 cells revealed a significant reduction in CCK efficacy (about 300 times lower) (Figure 1Bh and Figure 4Bb). Such an arrangement would probably minimize or even rule out any effect exerted by endogenous CCK or gastrin if miniSOG-CCK2R is expressed in vivo, whereas the photogenetic/photodynamic activation of miniSOG-CCK2R is readily executed by light irradiation. This would definitely be helpful in elucidating or authenticating any roles that CCK2R may play in vital physiological functions.

## 4. Discussion

In the present work, it was found that CCK2R expressed ectopically in CHO-K1 cells was fully functional and coupled to the calcium signaling pathway. The expressed CCK2R was permanently activated in the dark by the commonly used photosensitizer SALPC and analogue AlPcS_2_ at nanomolar concentrations, but not by central Al^3+^-free PcS_4_. Photodynamic action both with the dark-ineffective (3 nM) and dark-effective (10 nM) concentrations of SALPC sensitized both SALPC-activated and resting CCK2R. The genetically encoded protein photosensitizer miniSOG, on the other hand, could photodynamically activate CCK2R permanently as a fusion construct (miniSOG-CCK2R), although CCK efficacy for CCK2R in the constructed miniSOG-CCK2R was much reduced.

As mentioned above, CCK2 receptor is distributed both in the CNS and in peripheral tissues and plays an essential role in vital physiological functions [1,8,9,27,43]. In in vitro studies, CCK2R is often expressed ectopically in cell lines such as CHO-K1, as has been done previously by others [28,29]. In the present work, CCK stimulation of CCK2R-CHO-K1 cells was found to trigger regular calcium oscillations, which were inhibited completely by the CCK2R antagonist YM 022 (Figure 1). CCK-induced CCK2R desensitization, especially after prolonged stimulation at higher CCK concentrations, could particularly be noted (Figure 1 and Figure 4). All of these data confirm that the CCK2R we expressed heterologously in CHO-K1 cells was the quintessential CCK2 receptor.

The phthalocyanine photosensitizer SALPC was found to have no effect on baseline calcium in parental CHO-K1 cells in the dark, but dose-dependently induced persistent calcium oscillations in CCK2R-CHO-K1 cells (Figure 2). SALPC is not a polypeptide (like CCK or gastrin) but a tetrapyrrolic macrocycle. CCK2R is known to have similar affinity toward cholecystokinin and gastrin, which share a C-terminus of Gly-Trp-Met-Asp-Phe-NH_2_ [44]. Some other polypeptide CCK2R agonists, such as BC197 [cyclo(D.Asp-Tyr(SO_3_H)-Nle-D.Lys)-Trp-Nle-Asp-Phe-NH_2_] [45], BC264 [Boc-Tyr(SO_3_H)-gNle-mGly-Trp-NMeNle-Asp-Phe-NH_2_] [46], and BBL454 [H-(CH_2_)_5_-NHCO-CH_2_-CO-Trp-NMe-Nle-Asp-Phe-NH_2_] [47], all share the common sequence of Trp-NMeNle-Asp-Phe-NH_2_.

Remarkably, in the present work, we found that not only SALPC (AlPcS_4_) but also AlPcS_2_ (although not PcS_4_) (which is macrocyclic and completely different from the common peptide structure) could permanently activate the ectopically expressed CCK2R in CCK2R-CHO-K1 cells in the dark to trigger persistent calcium oscillations (Figure 2). Since AlPcS_2_ and SALPC were effective in the dark, whereas PcS_4_ was not, it could be established that the central conjugated Al^3+^ was required, but only two peripheral sulfonate groups were sufficient for dark agonistic activity. The presence of the central diamagnetic Al^3+^ in the sulfonated phthalocyanine was essential for this dark agonistic activity. It may be noted here that the central conjugated Al^3+^ increased the excited triplet lifetime and both the triplet state and ^1^O_2_ quantum yields (ΦΔ ≥ 0.7) of the phthalocyanine photosensitizers [48]. However, the dark agonist activity of SALPC and AlPcS_2_ toward CCK2R may not be related to the extended triplet lifetime, the quantum yield of the triplet state, or the quantum yield of ^1^O_2_, since such dark effects do not require light irradiation.

Other nonpeptide CCK2R agonists have also been reported before, such as triazolobenzodiazepinones [49,50]. The phthalocyanine agonists (SALPC and AlPcS_2_) discovered in the present work represent a completely new class of CCK2R agonists with very long-lasting effects.

Relevant to the CCK2R agonistic activity of SALPC (AlPcS_4_) and AlPcS_2_, tetrakis-(di-isopropyl-guanidino) zinc phthalocyanine (Zn-DIGP), a phthalocyanine molecule with a central conjugated Zn^2+^, has recently been found to be an antagonist against the calcium-mobilizing G-protein-coupled receptor CXCR3, but at higher concentrations (IC_50_ of 3.8 µM) [51]. In the case of Zn-DIGP, its peripheral hydrophilic di-isopropyl-guanidino groups have been found to be essential to its dark antagonistic activity, but the central conjugated zinc was found not to be essential. Instead, the removal of central conjugated Zn^2+^ rather drastically enhanced its dark antagonist activity [51]. Interestingly, in a separate report, it was found that a completely water-insoluble gallium-conjugated phthalocyanine without any peripheral substitutions (GaPc) could be bound and dissolved by the bacterial heme acquisition system (Has) protein A (HasA) transported via HasA receptors into *Pseudomonas aeruginosa* bacterial cells for subsequent photodynamic inactivation [52]. The recognition of SALPC by CCK2R, Zn-DIGP by CXCR3, and Ga-Pc by HasA all clearly indicate that phthalocyanines *per se* in the complete absence of light irradiation are an important class of bioeffective molecules.

To examine the SALPC photodynamic effect, CCK2R-CHO-K1 cells were exposed to a low-dark ineffective concentration of SALPC at 3 nM, and subsequent red-light (>580 nm, 31.5 mW·cm^−2^, 5 min) irradiation was found to sensitize the CCK stimulation of CCK2R in CCK2R-CHO-K1 cells (Figure 3). After exposure to the lowest dark-effective concentration of SALPC 10 nM, which by itself elicited calcium oscillations, subsequent red-light illumination (>580 nm, 31.5 mW·cm^−2^, 5 min) markedly enhanced the oscillatory frequency of SALPC 10 nM-induced calcium oscillations (Figure 3). These data, altogether, suggest that SALPC photodynamic action sensitizes both CCK2R activated by low amounts of SALPC and resting CCK2R in CCK2R-CHO-K1 cells.

Other than CCK2R sensitization through SALPC photodynamic action, we also examined photodynamic CCK2R modulation with the genetically encoded protein photosensitizer miniSOG. miniSOG (106 residues) with a noncovalently bound fluorophore flavin mononucleotide (FMN) is a protein photosensitizer with sufficient ^1^O_2_ quantum yield (≥0.03) [53,54]. Other than generating ^1^O_2_, miniSOG, upon excitation with blue light (448 nm), also emits green (500 nm) fluorescence [55,56] and therefore can be used as a fluorescent tag for protein subcellular localization and protein-protein interactions [57,58,59,60,61].

In the present work, miniSOG was target-expressed to the plasma membrane as a fusion construct, miniSOG-CCK2R, in CHO-K1 cells (Figure 4). Here, miniSOG photodynamic action with blue LED-light irradiation (450 nm, 85 mW·cm^−2^, 90 s) was found to trigger persistent calcium oscillations, which were completely blocked by the CCK2R antagonist YM 022 at 30 nM (Figure 4). At this concentration, YM 022 also blocked calcium oscillations induced by CCK 30 pM (Figure 1). LED light irradiation (450 nm, 85 mW·cm^−2^, 90 s) had no effect on baseline calcium in CCK2R-CHO-K1 cells expressing no miniSOG. In addition, miniSOG-CCK2R-CHO-K1 cells showed a very stable baseline calcium level in the absence of light irradiation. All of these data provide an unambiguous line of evidence that miniSOG photodynamic action permanently activates CCK2R in miniSOG-CCK2R-CHO-K1 cells (Figure 4).

The above data, altogether, indicate that photodynamic action with SALPC or with miniSOG either sensitizes or permanently activates CCK2R. Since SALPC binding to CCK2R is likely to alter its spatial conformation (an SALPC-activated state at SALPC 10 nM, or a meta-activated state at SALPC 3 nM), under these conditions, CCK2R could only be further sensitized by SALPC photodynamic action (Figure 3). However, for photodynamic action with the in-frame miniSOG of miniSOG-CCK2R, miniSOG photodynamic action acted on CCK2R, which was not in an activated but rather a resting state (or rather in an inhibited state—see the much reduced CCK affinity in miniSOG-CCK2R in Figure 4); therefore, CCK2R was permanently activated by miniSOG photodynamic action (Figure 4). Photodynamic sensitization or permanent activation as it might be, the relevant photooxidative chemistry could possibly account for such actions on CCK2R.

**Figure A1:**
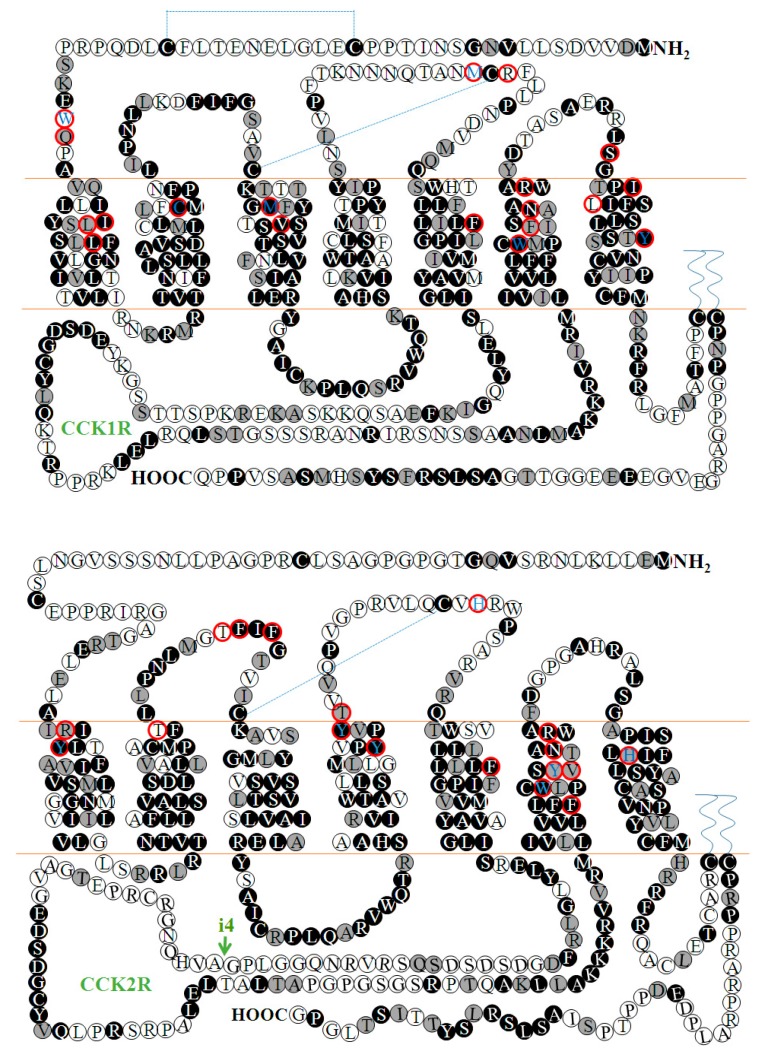
Plasma membrane topology of hCCK1R (upper panel) and hCCK2R (lower panel). Black circles filled with white letters indicate identical residues, gray circles indicate homologous residues. The dashed lines indicate disulfide bonds. Residues essential for agonist binding or CCK2R activation are in red boxes [69]. Blue letters indicate residues that play a key role in receptor activation and readily oxidized by ^1^O_2_ [70]. The green arrow in the lower panel indicates a site where a sequence of 69 residues is inserted due to a mis-spliced intron 4 [79].

Met, Cys, His, Trp, and Tyr residues are ready oxidative targets for ^1^O_2_ generated in type II photodynamic actions [32,62,63]. Met oxidation is especially known to modulate protein activities and related cellular functions [64]. CCK1R is the first reported G-protein-coupled receptor to be permanently activated by ^1^O_2_ generated in photodynamic action with photosensitizers SALPC, KillerRed, and miniSOG [32,41,42]. Although every detail of the molecular mechanisms and oxidant chemical bases for such permanent activation are not yet known, a comparison of residues key to the activation of CCK1R and CCK2R (but also susceptible to ^1^O_2_ oxidation) might give some useful hints. Out of the 447 residues in the full CCK2R sequence, the following are susceptible to ^1^O_2_ oxidation: 10 Met residues—Met1, Met67, Met73, Met108, Met117, Met134, Met186, Met234, Met334, and Met393; 15 Cys residues—Cys22, Cys39, Cys107, Cys127, Cys157, Cys205, Cys279, Cys293, Cys345, Cys384, Cys391, Cys401, Cys405, Cys408, and Cys409; 6 His residues—His170, His207, His274, His364, His376, and His394; 6 Trp residues—Trp165, Trp179, Trp209, Trp218, Trp346, and Trp355; and 12 Tyr residues—Tyr61, Tyr132, Tyr153, Tyr189, Tyr192, Tyr238, Tyr246, Tyr294, Tyr350, Tyr380, Tyr390, Tyr438, Tyr61, Tyr189, Tyr192, and Tyr350 (Figure A1 in Appendix A). The CCK2R residues that have been found to be essential for CCK binding (as determined by site-directed mutagenesis and photoaffinity labeling) are as follows: Arg57, Tyr61, Thr111, Thr119, Phe120, Phe122, Tyr189, Tyr192, Thr193, His207, Phe227, Phe342, Trp346, Val349, Tyr350, Asn353, Arg356, and His376 [65,66,67,68,69,70,71]. The overlapping residues in CCK2R are *Tyr61, Tyr189, Tyr192, His207, Trp346, Tyr350,* and *His376*. As a comparison, the CCK1R residues key to CCK1R activation but also oxidizable by ^1^O_2_ are *Trp39, Cys94, Met121, Met195, Trp326,* and *Tyr360* [69,70,72,73,74]. The similarities between the two groups of overlapping (critical for receptor activation but also susceptible to ^1^O_2_ oxidation) residues may not be immediately apparent. It may be noted, however, that CCK2R and CCK1R share a conserved E/DRY motif at the junction of TM-3 and ICL-2 and an NPxxY motif at the junction of TM-7 and the C-terminus tail (Figure A1). Both motifs are important for CCK receptor activation. The residue Y in E/DRY and NPxxY is readily oxidized by ^1^O_2_.

It may be noted here that although in the present work calcium mobilization was used to gauge CCK2R activation both by CCK and by ^1^O_2_, CCK2R is known to be coupled to multiple signaling pathways. Other than the Gq–phospholipase C signaling pathway, CCK2R (in biased signaling) is typically also coupled to the β-arrestin signaling pathway. Importantly, the CCK2R recruitment of β-arrestin 2 signaling has been found to be critically dependent upon the sulfur–aromatic interaction between Met^134^ (Met^3.32^) and Tyr^380^ (Tyr^7.43^) [75]. Note that both Met^134^ and Tyr^380^ are readily oxidized by ^1^O_2_, and therefore their oxidation might modulate β-arrestin recruitment by CCK2R. The β1 adrenergic receptor recruitment of β-arrestin 2 signaling has actually been directly linked to internal calcium release after SUMOylation of sarcoplasmic reticulum Ca^2+^-ATPase [76].

**Figure 5:**
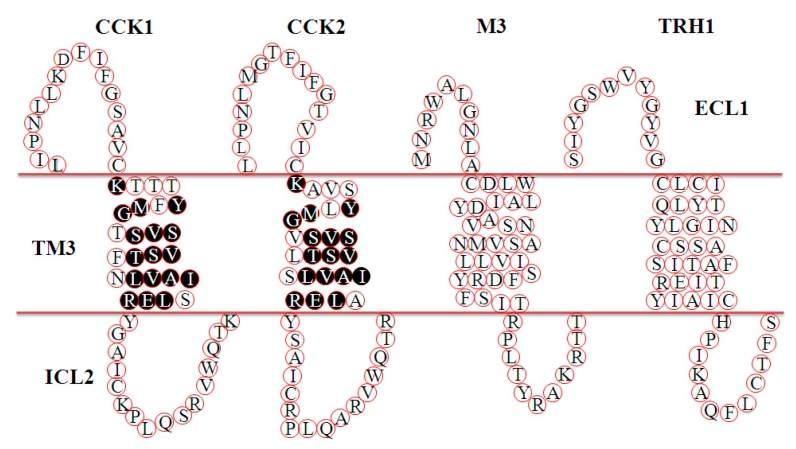
Comparisons of TM3 in CCK1R, CCK2R, M3R, and TRH1R. The TM3 sequences of CCK1R and CCK2R are from Reference [70], and the sequences of M3R and TRH1R are from the National Library of Medicine database (https://www.ncbi.nlm.nih.gov/pubmed) (NP_001334645.1 and NP_003292.1, respectively). M3R: M3 acetylcholine receptor; TRH1R: thyrotropin releasing hormone receptor 1; TM3, transmembrane domain 3; ECL1: extracellular loop 1; ICL2: intracellular loop 2.

The amino acid sequences of hCCK1R and hCCK2R are 53% identical in the full sequence and 69% identical in the TM segments (Figure A1). Out of the seven TM domains, we have previously found that TM3_CCK1R_ is the pharmacophore responsible for the miniSOG photogenetic activation of CCK1R [42]. A comparison of the TM3 domains of ^1^O_2_-activated CCK1R (*K*TTT*Y*F*MG*T*SVSVST*FN*LVAI*S*LER*) and CCK2R (*K*AVS*Y*L*MG*V*SVSVST*LS*LVAI*A*LER*) and ^1^O_2_-nonsusceptible M3 [39] (CDLWLAIDYVASNASVMNLLVISFDRYFSIT) and TRH1 (Jin, Cui, unpublished) (CLCITYLQYLGINASSCSITAFTIERYIAIC) revealed that the sequence of YxM (x for any residue) in *K*xxx*Y*x*MG*x*SVSVST*xx*LVAI*x*LER* might be important in the ^1^O_2_ activation of CCK1 and CCK2 receptors (Figure 5).

Evidence of the permanent activation of CCK2R is rather scarce in the literature. A mis-spliced form of CCK2R retaining intron 4 (corresponding to an insertion of 69 amino acid residues in the middle of intracellular loop 3—ICL3, Figure A1) and expressed in stomach, colon, and pancreatic cancers [10,11,77] has been shown to be more effective compared to the wild type in mobilizing cytosolic calcium expressed in CHO cells [78]. The expression of this ICL3-expanded version of CCK2R in Balb3T3 cells was found to result in constitutive CCK2R activation and spontaneous calcium oscillations not blocked by the CCK2R antagonist CAM1028 [79], but such constitutive CCK2R activation and spontaneous calcium oscillations could not be confirmed after expression in CHO cells [78]. Another case of constitutively active CCK2R is mutant CCK2R^E151A^ (E151 of the E/DRY motif) accompanied by enhanced cell proliferation and invasion [67], but this mutation (E151A) is obviously not related to the photooxidative activation of CCK2R since the ^1^O_2_-susceptible residue Y remains. Future elucidation on the three-dimensional structure of resting and agonist-activated CCK2R may shed light on uncertainties with regard to the atomic details of permanent photooxidative CCK2R activation. Such works might also resolve the difference between reversible and permanent CCK2R activation through agonists or photooxidation, respectively.

CCK2R is the second G protein-coupled receptor activated by singlet oxygen (GPCR-ABSO). In future in vivo applications, photodynamic CCK2R activation could potentially be done with knocked-in miniSOG or other genetically encoded protein photosensitizers. The permanent photooxidative activation of CCK2R may be used to verify CCK2R functions both in the CNS and in peripheral tissues or to actuate CCK2R functions in expressing and nonexpressing cell types after the knocked-in expression of the construct miniSOG-CCK2R. In such a scenario, interference from endogenous CCK or gastrin might be rather limited because of the much reduced affinity of miniSOG-CCK2R toward endogenous agonists in comparison to the free-standing CCK2R (compare Figure 1 and Figure 4). Such a technique might also be used to relieve neurogenic pain or to eradicate CCK2R-bearing cancer cells. It may be noted here that type II photodynamic action is also found with the endogenous photosensitizers riboflavin, flavin mononucleotide (FMN), flavin adenine dinucleotide (FAD) present in the skin [80]. The absorption of sunlight in the ultraviolet A region (UVA) by any CCK2R-expressing cells present in the skin might well be activated by UVA exposure.

## 5. Conclusions

**Figure 6:**
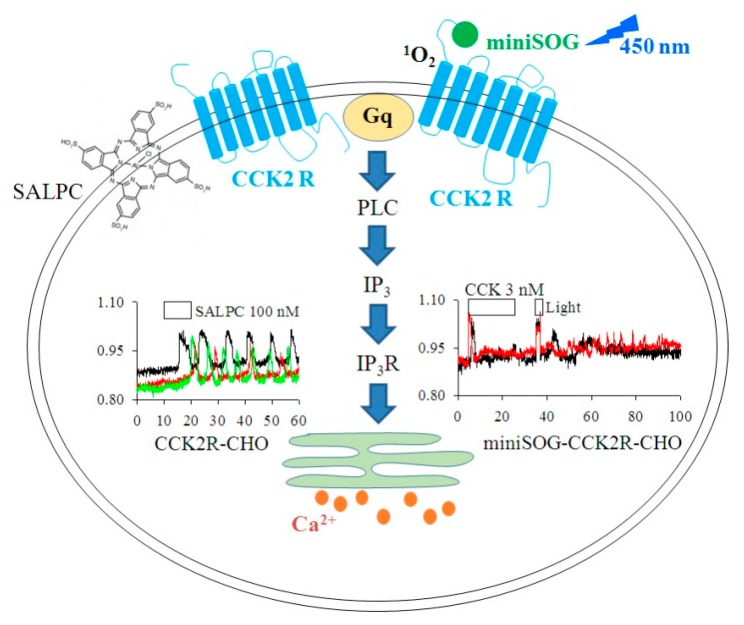
SALPC in the dark and miniSOG photodynamic action permanently activated CCK2R. (1) SALPC in the dark as an agonist for CCK2R to trigger persistent calcium oscillations in CCK2R-CHO-K1 cells. (2) The miniSOG photodynamic activation of CCK2R (miniSOG-CCK2R) elicited persistent calcium oscillations in miniSOG-CCK2R-CHO-K1 cells. Gq, G protein; PLC, phospholipase C; IP_3_R, IP_3_ receptor/channel complex; ER, endoplasmic reticulum. SALPC irradiated with light > 580 nm and miniSOG at 450 nm. ^1^O_2_ was produced in photodynamic action with miniSOG.

In conclusion, SALPC is a novel CCK2R agonist with long-lasting effects, and miniSOG photodynamic action activates CCK2R permanently (Figure 6). The long-lasting or permanent activation of CCK2R by SALPC in the dark or through miniSOG photodynamic action after miniSOG fusion to the N-terminus of CCK2R could both be applied to verify the in vivo functions of CCK2R in multiple types of cells or tissues, and to potentially treat pain or cancer.
